# Elevated exposures to persistent endocrine disrupting compounds impact the sperm methylome in regions associated with autism spectrum disorder

**DOI:** 10.3389/fgene.2022.929471

**Published:** 2022-08-11

**Authors:** Angela G. Maggio, Henry T. Shu, Benjamin I. Laufer, Chongfeng Bi, Yinglei Lai, Janine M. LaSalle, Valerie W. Hu

**Affiliations:** ^1^ Department of Biochemistry and Molecular Medicine, The George Washington University School of Medicine and Health Sciences, Washington, DC, United States; ^2^ The Johns Hopkins University, School of Medicine, Baltimore, MD, United States; ^3^ Genome Center, Perinatal Origins of Disparities Center, Environmental Health Sciences Center, Medical Microbiology and Immunology, MIND Institute, UC Davis School of Medicine, Davis, CA, United States; ^4^ Department of Statistics, The George Washington University, Washington, DC, United States

**Keywords:** endocrine disrupting compounds, DNA methylation, sperm, Faroe Islands, autism

## Abstract

Environmental exposures to endocrine disrupting compounds (EDCs) such as the organochlorines have been linked with various diseases including neurodevelopmental disorders. Autism spectrum disorder (ASD) is a highly complex neurodevelopmental disorder that is considered strongly genetic in origin due to its high heritability. However, the rapidly rising prevalence of ASD suggests that environmental factors may also influence risk for ASD. In the present study, whole genome bisulfite sequencing was used to identify genome-wide differentially methylated regions (DMRs) in a total of 52 sperm samples from a cohort of men from the Faroe Islands (Denmark) who were equally divided into high and low exposure groups based on their serum levels of the long-lived organochlorine 1,1-dichloro-2,2-bis(p-chlorophenyl)ethylene (DDE), a primary breakdown product of the now banned insecticide dichlorodiphenyltrichloroethane (DDT). Aside from being considered a genetic isolate, inhabitants of the Faroe Islands have a native diet that potentially exposes them to a wide range of seafood neurotoxicants in the form of persistent organic pollutants (POPs). The DMRs were mapped to the human genome using Bismark, a 3-letter aligner used for methyl-seq analyses. Gene ontology, functional, and pathway analyses of the DMR-associated genes showed significant enrichment for genes involved in neurological functions and neurodevelopmental processes frequently impacted by ASD. Notably, these genes also significantly overlap with autism risk genes as well as those previously identified in sperm from fathers of children with ASD in comparison to that of fathers of neurotypical children. These results collectively suggest a possible mechanism involving altered methylation of a significant number of neurologically relevant ASD risk genes for introducing epigenetic changes associated with environmental exposures into the sperm methylome. Such changes may provide the potential for transgenerational inheritance of ASD as well as other disorders.

## Introduction

Environmental exposures to endocrine disrupting compounds (EDCs) have been linked with various diseases including neurodevelopmental disorders (Gore et al., 2014; Schug et al., 2015; Braun, 2017; Rivollier, Krebs and Kebir, 2019). Autism spectrum disorder (ASD) is a highly complex neurodevelopmental disorder characterized by deficits in social communication, repetitive behaviors, and restricted interests which has increased in prevalence in the United States from roughly 1 in 125 children in 2004 to 1 in 54 children in 2016 (Maenner et al., 2020; American Psychiatric Association Task Force on DSM-5, 2013). Although ASD exhibits a strong genetic component as revealed by higher concordance rates in affected monozygotic twins in comparison to dizygotic twins (Hallmayer et al., 2011; Tordjman et al., 2014) coupled with the identification of genetic variants in now over a thousand genes (Abrahams et al., 2013), the incomplete penetrance of ASD even in monozygotic twins and the limited effect sizes of the majority of genetic variants also suggest a role for environmental risk factors or gene by environment interactions.

Experimental and epidemiological studies have reported harmful effects of environmental exposures to both short- and long-lived EDCs, such as the short-lived bisphenol A (BPA) and phthalates, and persistent organic pollutants (POPs) like polychlorinated biphenyl (PCB) and polybrominated diphenyl ether (PBDE) compounds, on social behavior and neurodevelopment. Rodent studies have shown associations between EDC exposure and disruption of social hormones, social recognition, locomotion, and excitatory-inhibitory synapse pathways (Gogolla et al., 2009; Wolstenholme et al.,2013; Lichtensteiger et al., 2015). Exposure to 3,3′-dichlorobiphenyl also exerted effects on axonal and dendritic growth in primary rat neurons (Sethi et al., 2017). At the genomic level, prenatal exposure of pregnant rats to BPA was associated with sex-dependent transcriptomic changes in both the hippocampus and prefrontal cortex of neonatal rats (Thongkorn et al., 2019; Kanlayaprasit et al., 2021; Thongkorn et al., 2021). Notably, the differentially expressed genes between the BPA-exposed and unexposed offspring were enriched in ASD risk genes (Kanlayaprasit et al., 2021). Epidemiological studies further show that prenatal human exposures to EDCs, specifically DDE and PCBs, have been linked to alterations in hormone levels in offspring with potential for lasting and widespread signaling disruption (Braun, 2017; Eskenazi et al., 2017).

With respect to mechanisms of action, many studies have investigated epigenetics as a potential mediator of EDC-induced changes in phenotype and disease state. Epigenetic mechanisms involve modification of gene expression or regulation that do not involve changes in DNA sequence. These changes may be due to DNA methylation, histone modification and microRNA induction or suppression. For example, Manikkam et al. (2012) showed that certain plastics-derived EDCs induced transgenerational inheritance of obesity, reproductive disease and epigenetic modifications in sperm. More recently, McBirney et al. (2017) reported that exposure of pregnant rats during gestation to the herbicide atrazine resulted in transgenerational transmission of diseases and body phenotypes as well as DNA methylation changes in sperm in the F1 through F3 offspring. Regarding EDC involvement in neurodevelopment, Dunaway et al. (2016) demonstrated that treatment of a neuronal cell model with PCB 95 was associated with significant global DNA hypomethylation of autism candidate genes with a direct impact on gene expression. In the same study, hypomethylation was observed in brain tissues from individuals with Dup15q syndrome (a condition frequently associated with ASD) which had been previously associated with exposure to PCB 95 (Mitchell et al., 2012). The role of epigenetics as an underlying mechanism for EDC-induced neurodevelopmental disorders including ASD is further discussed in a number of recent reviews (LaSalle, 2013; Bakulski et al.2014; Tran and Miyake, 2017; Moosa et al., 2018).

In addition to having an impact on neurodevelopment, EDCs have also been implicated in many other diseases or human conditions, such as cancer, diabetes, obesity, metabolic disorders, and infertility (De Coster and Van Larebeke, 2012). With respect to infertility, human exposures to a variety of organochlorines have been shown to affect chromatin integrity (Rignell-Hydbom et al., 2005) and sex chromosome aneuploidy in sperm (McAuliffe et al., 2012). EDC-associated sex chromosome aneuploidy in sperm was further confirmed in an additional study involving a cohort of men in the Faroe Islands who have higher than average exposures to POPs based on their diet which is rich in pilot whale meat and blubber that can serve as a reservoir for lipophilic POPs (Perry et al., 2016).

In recent years, the sperm methylome (i.e., genome-wide DNA methylation pattern) has come under increasing scrutiny as a source of epigenetic changes that may have an impact on a wide variety of human disorders, from infertility to neurodevelopmental and psychiatric disorders (Wu et al., 2015b). DNA methylation changes in sperm have been reported as a function of advanced paternal age in both mice and men (Jenkins et al., 2014; Milekic et al., 2015). Notably, the changes have included genes that overlap with those reportedly associated with autism, schizophrenia, and bipolar disorder. Since advanced paternal age has also been associated with risk for autism (Reichenberg et al., 2006; Vierck and Silverman, 2014; Quinlan et al., 2015), Feinberg et al. investigated DNA methylation differences in sperm from fathers with a child with ASD in comparison to that from fathers of neurotypical children (Feinberg et al., 2015). They identified 193 DMRs in sperm of fathers with children affected by ASD; many of these DMRs were in the proximity of genes involved in developmental processes. A more recent study on the sperm methylome of fathers who have children with or without ASD confirmed methylation differences in sperm and further demonstrated the potential for development of a biomarker screen for ASD based on DMRs (Garrido et al., 2021). These studies thus establish a link between DNA methylation changes in sperm and possible risk for ASD in children of fathers that exhibit such methylomic modifications. A still unanswered question concerns the mechanism through which these methylomic changes in sperm may occur.

Based on the above studies, we postulated that elevated exposures to EDCs is associated with methylomic changes in sperm DNA in regions that may influence risk for ASD. The primary objectives of this study are to: 1) determine if high versus low exposures to the persistent organochlorine 1,1-dichloro-2,2-bis(p-chlorophenyl)ethylene (DDE) as determined by serum levels are associated with DMRs in sperm from a Faroese cohort whose native diet, often rich in pilot whale meat and blubber, may expose a fraction of the individuals to higher than average amounts of EDCs; 2) determine the nature of genes associated with such DDE DMRs; 3) identify pathways and functions over-represented among DMR-associated genes.

## Materials and methods

### Study design

**FIGURE 1:**
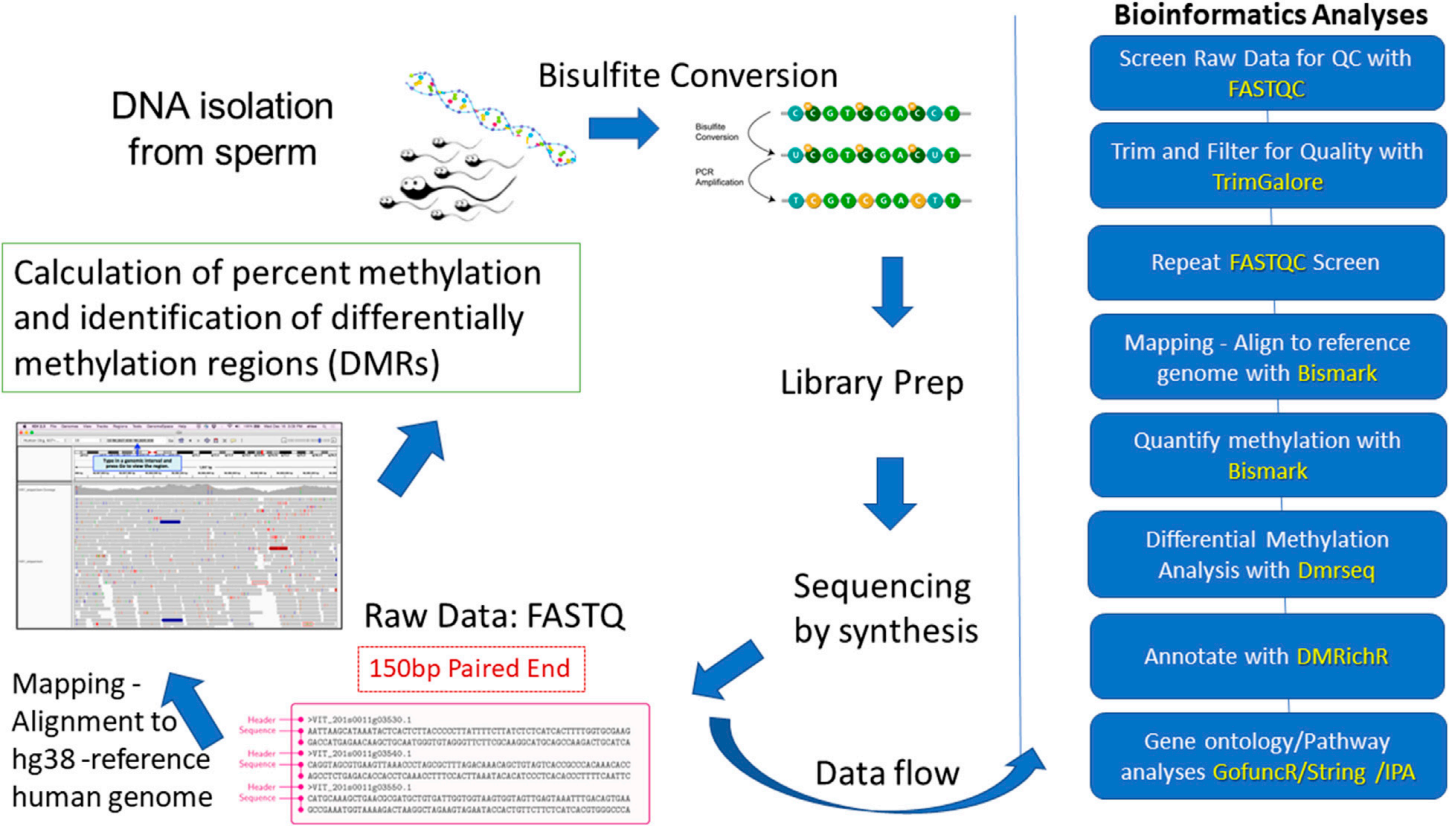
Overview of workflow for this study. The blue blocks on the right outline the analytical workflow and programs (identified by yellow font) used to identify and characterize the DMRs.

The overall workflow for this study is summarized in Figure 1. Here, we used whole genome bisulfite sequencing (WGBS) to investigate differences in DNA methylation in sperm from 52 men exhibiting the highest (*n* = 26) and lowest (*n* = 26) exposures to DDE, a stable breakdown product of the insecticide DDT. The highest and lowest exposures were determined by the levels of DDE that were quantitated in the serum of semen donors. The serum levels were divided into tertiles, with the highest tertile representing the high exposure group, and with the lowest tertile representing the low exposure group which served as the “control” group for this study. Serum levels of DDE within the first tertile ranged from 1 to 249 ng/g lipid while those within the third tertile ranged from 578 to 3,328 ng/g lipid. For comparison, a recent meta-analysis of blood levels of DDE across the globe after the year 2000 shows an average blood level of 207 ng/g lipid (Koureas et al., 2019) which is comparable to levels within the first tertile of our study. It should be noted that all 52 semen samples from the combined exposure groups were processed blindly through the bisulfite sequencing step to avoid any experimental bias in the handling of the samples. After the bisulfite sequencing was completed, the blind was broken and samples were randomly divided into discovery (*n* = 32) and validation sets (*n* = 20) for WGBS analyses of the methyl sequencing data. The purpose of using discovery and validation sample sets in this study was to assess the reproducibility of the DMRs identified by the methyl-seq analyses. Gene ontology and pathway analyses were employed to determine enrichment in pathways and functions among the DMR-associated genes. Differential methylation of selected genes relevant to ASD and other neurodevelopmental disorders was further validated by pyrosequencing analyses.

### Semen samples and demographics of donors

The semen donors whose de-identified samples were included in this study were among several hundred young men recruited from the general population register of individuals born in the Faroe Islands (Denmark) between 1981 and 1984. Samples were collected throughout 2007 and 2008 and de-identified by the Faroese Hospital System. Information related to height, weight, age, smoking status, sample collection date, length of abstinence at the time of collection, and the serum concentrations of 13 persistent organic pollutants (POPs) including the four most prevalent PCB congeners, (PCB 118, PCB 138, PCB 153, PCB 180) as well as DDT and DDE were recorded for each sample as part of the Faroese General Population research cohort. This cohort was part of a broader parent study on Faroese health and fertility that was approved by the local Science Ethics Committee for the Faroe Islands. Thus, the sample data also included information on sperm concentration and motility levels, both of which have relevance to male fertility. Raw ejaculate (i.e., semen) from 52 young men in this cohort was kindly provided for this specific study through a Data Processor Agreement between Dr. Pál Weihe, Director of the Department of Occupational Medicine and Public Health of the Faroese Hospital System in Tórshavn, Faroe Islands and Dr. Valerie Hu at The George Washington University. The advantage of using the Faroese cohort for this study on the sperm methylome in the context of environmental exposures to EDCs is that inhabitants of the Faroe Islands are considered genetic isolates and therefore are expected to have similar genetic background and polymorphisms that would be considered major confounding variables in genomics studies. In addition to the de-identified semen samples, Dr. Weihe also provided the demographic data and serum levels of the POPs that had been determined for each donor as previously described (Grandjean et al., 2001). Faroese semen samples with a minimum sperm count of 20 × 10^6^ per ml were divided by Dr. Melissa Perry (Dept. of Environmental and Occupational Health, GWU) into three exposure groups based on the recorded levels of DDE in serum, and 26 samples each from the first (lowest exposure) and third (highest exposure) tertiles were provided for this methylation study without assignment of the samples to exposure tertiles. Thus, the semen samples were processed through bisulfite sequencing analysis without knowledge of their respective exposure levels.

### Sperm isolation and DNA extraction methods

Sperm was isolated from semen (which contains various cell types) using a published discontinuous gradient protocol (Wu et al., 2015a). Briefly, 100 μl of semen was washed with 2 ml Quinn’s Sperm Washing Medium (Origio, Trumbull, CT, United States), and cells were pelleted at 600 g for 5 min at 4°C. The pelleted cells were resuspended in 0.5 ml Quinn’s solution and counted in a hemocytometer to determine the initial number of sperm and somatic cells in the semen sample. Next, a discontinuous gradient of PureCeption solution (Origio) diluted with Quinn’s was formed in a 15 ml conical tube with 1.5 ml of 40% PureCeption over 1.5 ml of 90% PureCeption. The washed cells were layered on top of the gradient before centrifugation at 300 g for 20 min at room temperature. The pellet was transferred to a new 15 ml tube and resuspended in 3 ml Quinn’s Washing solution. The cells were pelleted at 600 g for 5 min, and then resuspended in 500 μl Quinn’s after removal of the supernatant. Cells were recounted to determine total number of sperm and somatic cell contamination (which never exceeded 1%) before centrifugation at 4,000 g for 1 min.

DNA was isolated from the purified sperm cells using a Qiagen AllPrep DNA/RNA mini kit following the manufacturer’s protocol. Sperm pellets were first lysed in 450 μl RLT buffer with 50 μl added tris(2-carboxyethyl)phosphine solution (TCEP, a bond-breaker) by vortexing with 0.1 gm RNase/DNase-free stainless steel microbeads for 5 min at RT in a Disruptor Genie (Scientific Industries, Bohemia, NY, United States). Lysates were immediately stored at −80°C until DNA extraction which was usually completed the next day using the standard protocol.

### Whole genome bisulfite sequencing

The sperm DNA samples were sent to Admera Health (South Plainfield, NJ, United States), a CLIA-certified laboratory, for WGBS analyses. The directional Illumina TruSeq DNA Methylation library kit was used for sample preparation. WGBS was then performed on an Illumina HiSeq X sequencer resulting in 150 bp PE reads achieving roughly 4x coverage genome wide per sample. Raw FASTQ files were received from Admera Health for further analyses.

### Whole genome bisulfite sequencing bioinformatics pipeline

We utilized a bioinformatics pipeline comprised of CpG_Me for alignment and DMRichR for differential methylation determination as published on github (https://github.com/ben-laufer). CpG_Me builds on previously published bioinformatic tools and pipelines (Krueger and Andrews, 2011; Laufer et al., 2019). The DMRichR workflow similarly builds on previously established bioinformatic packages such as dmrseq and bsseq (Hansen et al., 2012; Korthauer et al., 2019; Laufer et al., 2019). All WGBS data was analyzed on The George Washington University’s high-performance cluster, Colonial One. First, raw read files (FASTA) were trimmed and quality checked with Trim-Galore and FASTQC software. Forward reads were trimmed by 8 bp on the 3′- and 5′-ends. Reverse reads were trimmed 8 bp on the 3′-ends and 20 bp on the 5′-ends in order to remove methylation bias often seen at the ends of reads. M-Bias plots from FASTQC analyses were examined to determine if trimming was sufficient. Trim Galore was also used to filter out bases with Phred scores lower than 20 that would indicate a 1 in 100 probability of an incorrect base call. Reads were then aligned to a reference human genome (hg38) using Bismark, a three-letter aligner. Methylation calls are differentiated among CpG, CHG, and CHH contexts (Krueger and Andrews, 2011), but only the CpG sites were considered in this study. Approximately 74% of bisulfite reads aligned to a bisulfite-converted reference human genome allowing for the assay of 10.08 million CpG sites. All raw and processed data from the WGBS analyses along with demographic data have been deposited into the NBCI’s Gene Expression Omnibus (GEO) repository (GEO Accession number GSE165915).

### Identification of differentially methylated regions

DMRs were identified using dmrseq and bsseq Bioconductor packages in the wrapped pipeline of DMRichR. Default parameters for the DMRichR executable script were used which included coverage set to 1x, per Group set to 100%, minCpGs set to 5, and maxPerms set to 10. Covariables adjusted for in DMR analysis included BMI, age, days in storage, batch effects of processor and date of processing, percent motile sperm, and smoking status. DMRs with permutation *p* ≤ 0.05 were identified for both discovery and validation sample sets. Differences in percent methylation (a measure of effect size) ranged from ±7 to ±38 for all DMRs, as shown in Supplementary Tables S1, S2. With DMRichR, DMRs were annotated for genomic and CG context. Annotation resources within DMRichR include the annotatr and rGREAT (Genomic Regions Enrichment of Annotations Tool) open source packages in Bioconductor. Annotatr (Cavalcante and Sartor, 2017) was used to visualize and compare annotated genomic sites/regions (e.g., promoters, 5′UTRs, exons, introns) that were identified within the discovery and validation sets, while rGREAT (Gu, 2014) was used for mapping genes to the sites. The mapped genes from each set were then utilized for pathway and functional analyses using Ingenuity Pathway Analysis software (Qiagen, Germantown, MD, United States) as described below.

### Gene ontology and network prediction analysis

Gene ontology analysis of the DMRs assigned to genes was accomplished using two different programs; GofuncR (Grote, 2020), which was modified for use with DMRichR, and the open-access STRING Version 11.0 (Search Tool for the Retrieval of Interacting Genes/Proteins) (Szklarczyk et al., 2019). GofuncR maps a DMR to a gene if it is between 5 kb upstream and 1 kb downstream of the gene body and also uses information from the background regions. A custom meta *p*-value analysis was performed using the sum of logs of the *p*-values of GO terms to integrate the data from the discovery and validation sets, and then terms with a meta *p*-value < 0.05 were slimmed using REVIGO (Supek et al., 2011) to reduce redundancy among the most significant GO terms. Ingenuity Pathway Analysis (IPA) Version 01–13 (Qiagen, Germantown, MD, United States) was used to discover pathways and functions enriched among the DMR-associated genes from each cohort (i.e., discovery and validation) based on Fisher Exact *p*-values of ≤ 0.05, using the curated genes in IPA’s Knowledgebase as the reference gene set.

### Pyrosequencing analyses

Pyrosequencing analyses of selected DMRs were performed in the Weksberg laboratory at The SickKids Research Institute of The Hospital for Sick Children; Toronto, Ontario, Canada. These DMRs included specific regions for *CSMD1, NRXN2, RBFOX1, PTPRN2, SNORD115-30,* and *SNORD115-37* that were identified by the WGBS analyses. Primers for each of the regions that typically included multiple methylation sites as well as the PCR conditions for the pyrosequencing analyses are provided in Supplementary File S1. The resulting pyrosequencing data were returned to us for further analyses of the methylation profiles of each region as a function of DDE serum concentration (μg/g lipid) for each sample.

### Hypergeometric distribution analyses

Hypergeometric distribution analyses were employed to determine the significance of DMR-associated gene overlap between the discovery and validation data sets as well as between DMR-associated genes and autism risk genes from the SFARI Gene Database (Abrahams et al., 2013). First, the overlapping genes were identified using a Venn diagram software program called Venny 2.1.0 https://bioinfogp.cng.csic.es/tools/venny/ (Oliveros, 2007). Significant overlap between the DMR-associated genes and SFARI genes was determined by hypergeometric distribution analyses using the CASIO Keisan Online Calculator <http://keisan.casio.com/exec/system/1180573201>, with significance determined by an upper cumulative Q-value of ≤ 0.05. These two programs were also used to identify significant overlap of DMR-associated genes from this study and those from other studies.

## Results

### Demographics of the Faroese cohort and relationships between organochlorine 1,1-dichloro-2,2-bis(p-chlorophenyl)ethylene and other endocrine disrupting compound levels and sperm parameters

**FIGURE 2:**
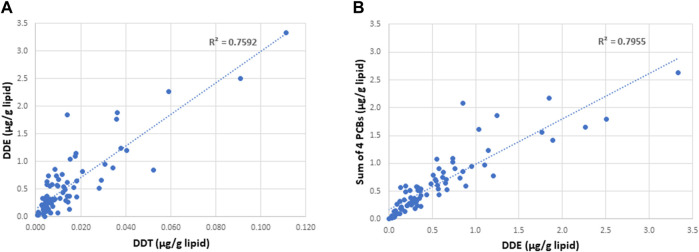
Correlation between the concentrations of DDE with those of DDT **(A)** and the sum of the four most prevalent PCB congeners **(B)** in the serum of semen donors. The 4 specific PCB congeners are PCB 118, PCB 138, PCB 153, and PCB 183.

**FIGURE 3:**
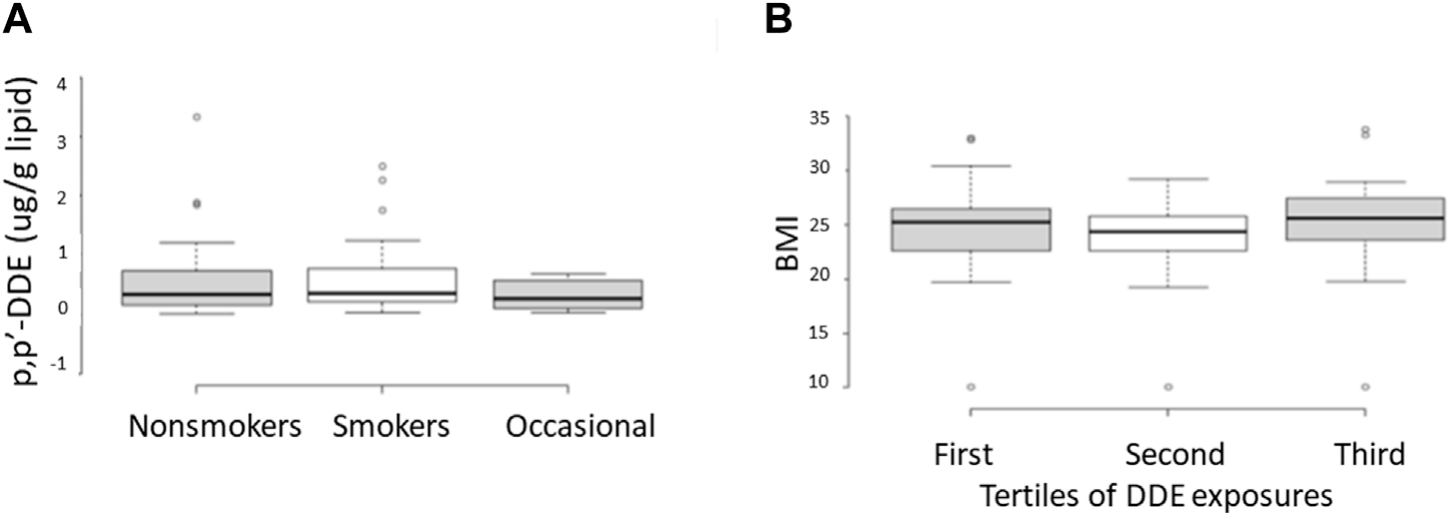
Relationship between serum concentrations of DDE and smoking status **(A)** or body mass index (BMI) **(B)**. In B, the range of BMIs is shown for individuals determined to be in the first, second, and third tertiles with respect to levels of serum DDE. Samples from the first and third tertiles were used to represent low and high exposures, respectively, in the current methylation analyses. These data show essentially no difference in the range of DDE exposures between smokers and nonsmokers as well as no difference in the range of BMIs as a function of DDE exposure levels.

**FIGURE 4:**
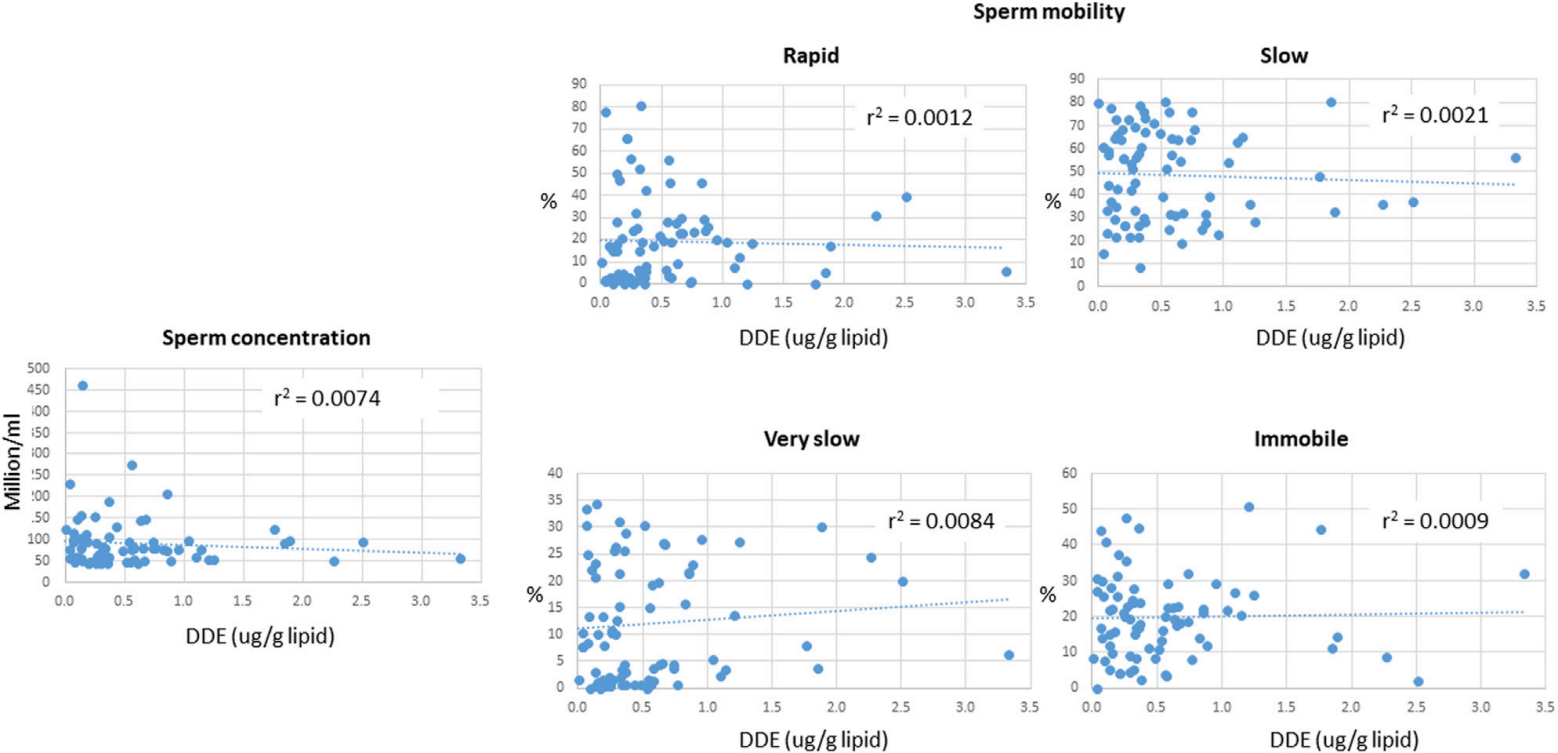
Relationship between serum concentrations of DDE and various sperm parameters, including sperm concentration and mobility. The motility terms “rapid”, “slow”, “very slow”, and “immobile” refer to the ability of sperm to move efficiently (which is required to reach an egg and achieve fertilization). The graphs show the % of sperm in each sample that exhibit the indicated motility as a function of DDE levels in serum.

A summary of demographic information and serum concentrations of EDCs for the cohort used in this study is provided in Supplementary Table S3. Regression analyses of the potential covariates were performed using statistical packages in Excel. These analyses showed that while there were strong correlations between the concentrations of DDE with those of DDT and the sum of the four most prevalent PCB congeners (PCB 118, PCB 138, PCB 153, PCB 180) (Figure 2), there were no significant correlations between DDE levels and smoking status, body mass index (BMI), or any of the measured sperm parameters, including sperm concentration and mobility level (Figures 3, 4).

### Differentially methylated regions associated with high versus low exposures to organochlorine 1,1-dichloro-2,2-bis(p-chlorophenyl)ethylene

**FIGURE 5:**
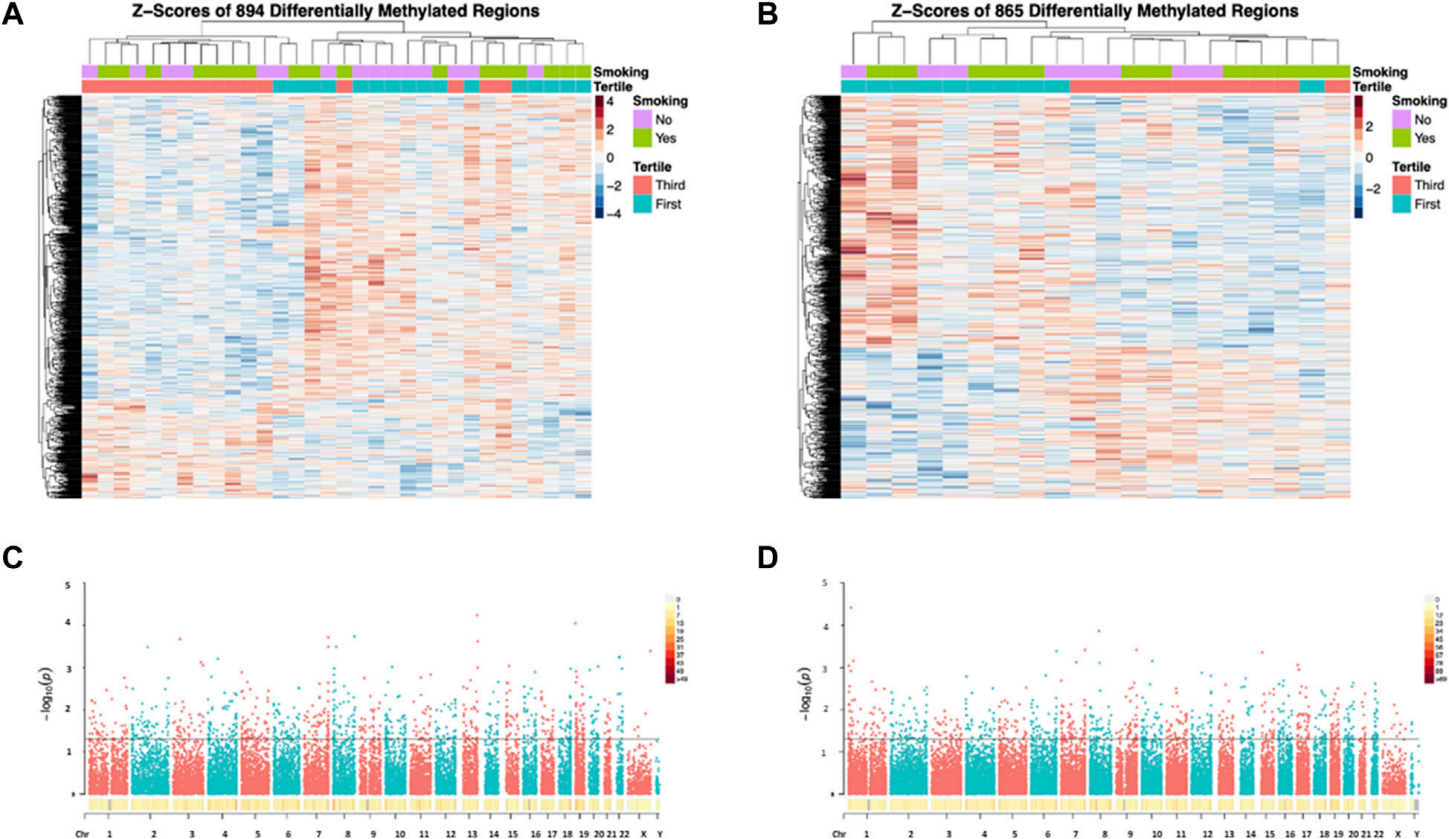
Heatmaps **(A,B)** and Manhattan plots **(C,D)** depicting the results of the WGBS analyses. DMRs from the Discovery set **(A)** and Validation set **(B)** are shown in the heatmaps. All significant DMRs are represented above the horizontal lines in the Manhattan plots (C = Discovery set; D = Validation set).

WGBS analyses of 32 samples included in the discovery set (with correction for all covariates including age, smoking status, BMI, sperm motility, days in storage, batch effects of processor and processing date) revealed a total of 894 DMRs (permutation *p* ≤ 0.05) between the first and third DDE exposure tertiles, while subsequent analyses of the validation set of 20 samples resulted in a total of 865 DMRs (Figure 5, DMRs in Supplementary Tables S1, S2).

**FIGURE 6:**
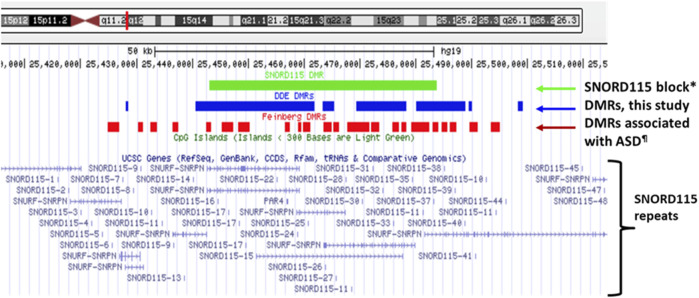
*SNORD115* block (*) on chromosome 15 showing significant genome-wide differential methylation between exposure groups after correction for multiple testing. The green bar identifies the location of the SNORD115 block located on chromosome 15, while the blue and red bars indicate the locations of the DMRs identified in this study and the prior study on sperm from fathers of children with ASD by Feinberg et al. (2015). The location of distinct SNORD115 repeats are also shown.

The overall distribution profiles of annotated gene and CpG regions were similar for both analyses (Supplementary Figure S1). Analysis of larger blocks of sequence for differential methylation revealed a single region that reached genome-wide significance after multiple testing correction (q < 0.022) when all 52 samples (26 with high exposure and 26 with low exposure) were combined (Figure 6). This block covered a 40,618 bp region of the *SNORD115* locus on chromosome 15 that is maternally imprinted, meaning expressed exclusively from the paternal allele. The validation data set shows nominal significance for increased methylation across this locus with a width of 63,606 bp (*p* < 0.011).

### Sperm differentially methylated regions associated with neurodevelopmental processes and ASD-risk genes

**FIGURE 7:**
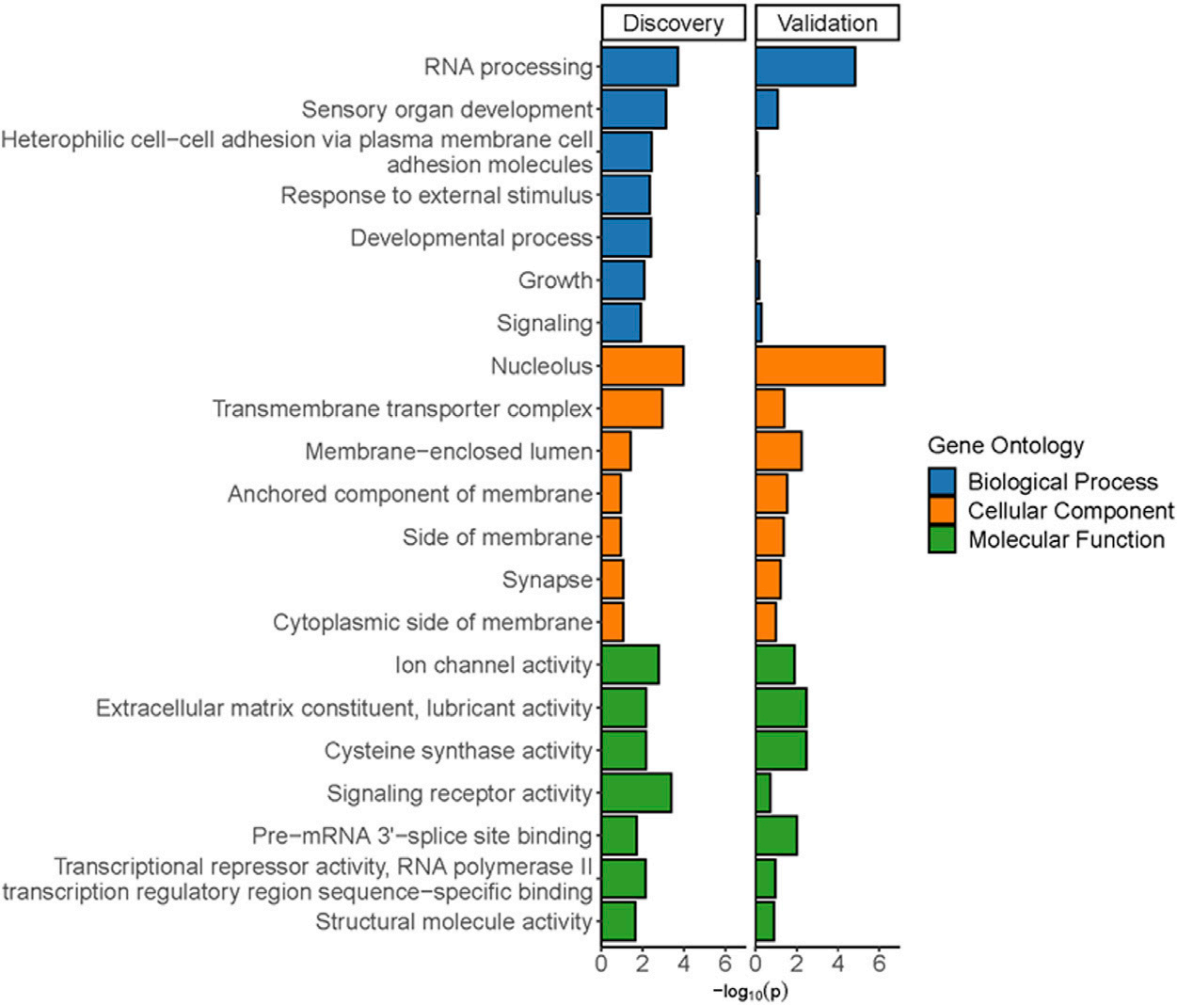
Significant gene ontology terms from GofuncR analyses of DMRs from discovery and validation cohorts. Specific *p*-values are from a meta *p*-value analysis of the least dispensable significant (*p* < 0.05) gene ontology terms.

Gene ontology analyses of the genes within the DMRs in the discovery and validation samples were performed using two different gene mapping and ontology approaches. GOfuncR analysis provides an overview of the top biological processes, cellular components, and molecular functions associated with the DMR-associated genes from each of the study cohorts (Figure 7), while STRING analysis of these genes not only replicates some of the GO terms from the GOfuncR analysis but also reveals significant over-representation of a number of processes involved in nervous system development and function that are shared by both data sets (Table 1). Notably, these shared processes include nervous system development, generation of neurons, neurogenesis, neuron differentiation, and synapse organization.

**TABLE 1:** Gene ontology terms enriched among DDE DMR-associated genes from discovery and validation cohorts as revealed by STRING analyses of each data set.

GO ID	Pathway description	Discovery FDR	Validation FDR
GO.0007399	Nervous system development	1.80E-11	4.80E-03
GO.0007275	Multicellular organism development	2.13E-08	1.60E-03
GO.0048731	System development	3.14E-08	4.10E-03
GO.0048856	Anatomical structure development	7.91E-08	2.20E-03
GO.0022008	Neurogenesis	5.51E-07	2.19E-02
GO.0032502	Developmental process	5.51E-07	5.50E-04
GO.0048699	Generation of neurons	5.51E-07	1.17E-02
GO.0048666	Neuron development	1.55E-06	4.12E-02
GO.0030182	Neuron differentiation	2.95E-06	2.19E-02
GO.0000904	Cell morphogenesis involved in differentiation	1.03E-05	3.89E-02
GO.0048468	Cell development	2.77E-05	1.48E-02
GO.0030154	Cell differentiation	1.50E-04	2.19E-02
GO.0048869	Cellular developmental process	2.50E-04	1.45E-02
GO.0048513	Animal organ development	2.80E-04	1.92E-02
GO.0003279	Cardiac septum development	7.10E-04	3.89E-02
GO.0050808	Synapse organization	1.80E-03	4.18E-02
GO.0072359	Circulatory system development	1.80E-03	4.12E-02
GO.0007155	Cell adhesion	2.40E-03	9.60E-03
GO.0003148	Outflow tract septum morphogenesis	3.30E-03	2.74E-02
GO.0050793	Regulation of developmental process	4.10E-03	2.60E-02
GO.0050794	Regulation of cellular process	5.80E-03	1.90E-04
GO.0000122	Negative regulation of transcription by RNA polymerase II	6.10E-03	7.70E-04
GO.2000026	Regulation of multicellular organismal development	6.10E-03	3.89E-02
GO.0050789	Regulation of biological process	1.04E-02	3.30E-04
GO.0006928	Movement of cell or subcellular component	1.50E-02	2.19E-02
GO.0007423	Sensory organ development	1.50E-02	1.05E-02
GO.0048518	Positive regulation of biological process	3.04E-02	3.80E-04
GO.0050767	Regulation of neurogenesis	4.36E-02	4.12E-02
GO.0051960	Regulation of nervous system development	4.54E-02	3.51E-02

The enrichment in neuronal processes was further confirmed by independent pathway and functional analyses of the DMR-associated genes from the discovery and validation cohorts using Ingenuity Pathway Analysis (IPA). CREB signaling in neurons, the endocannabinoid developing neuron pathway, netrin signaling, calcium signaling, GABA signaling, and axon guidance signaling are among the canonical pathways significantly enriched in both data sets (Table 2), while recognition of neurons, outgrowth of neurites, and neurotransmission are shared functions over-represented among the DMR-associated genes in each data set (Table 3).

**TABLE 2:** Canonical pathways enriched among DDE DMR-associated genes from discovery and validation cohorts.

Canonical pathways (discovery)	−Log (*p*-value)*	Molecules
CREB signaling in neurons	6.18	CACNA1I, CACNG6, GRID2, GRIA1, GRIK3, GNG2, FLT3, CACNB4, PIK3C2G, GNAI1, FGFR2, CREB5, GNG7, CACNA1A, GRM5, SHC1, GNAO1, IRS2, PRKCH, GRIK2, GRIK1
Glutamate receptor signaling	3.85	GRM5, GRID2, GRIA1, GNG2, GRIK3, GRIK2, GNG7, GRIK1
Endocannabinoid developing neuron pathway	3.65	FLT3, GNAO1, GNG2, GNAI1, PIK3C2G, FGFR2, PAX6, IRS2, GSK3B, CREB5, CTNNB1, GNG7
Netrin signaling	3.4	CACNA1I, NCK2, CACNG6, UNC5A, CACNB4, RYR3, NFATC1, CACNA1A
Calcium signaling	2.91	CACNA1I, CACNG6, MYH10, HDAC4, MYH13, GRIA1, CACNB4, TRPC4, CREB 5,NFATC1, CACNA1A, RYR3, SLC8A1, GRIK1
G beta gamma signaling	2.77	CACNA1I, CACNG6, SHC1, GNAO1, CACNB4, GNG2, GNAI1, PRKCH, GNG7, CACNA1A
Integrin signaling	2.6	TSPAN5, ITGA8, FLT3, PIK3C2G, TSPAN2, FGFR2, TNK2, ITGAL, NCK2, SHC1, IRS2, GSK3B, CTTN, NEDD9
Axonal guidance signaling	2.42	MMP20, LRRC4C, UNC5A, NTN4, GNG2, FLT3, PIK3C2G, GNAI1, SEMA6B, FGFR2, SLIT2, DPYSL5, PDGFC, GNG7, NFATC1, NCK2, SHC1, SEMA6D, NTNG2, GNAO1, IRS2, PRKCH, GSK3B, SEMA3C
Androgen signaling	2.37	CACNA1I, CACNG6, SHC1, GNAO1, CACNB4, GNG2, GNAI1, PRKCH, GNG7, CACNA1A
Relaxin signaling	2.31	PDE10A, RXFP1, FLT3, GNAO1, GNG2, GNAI1, PIK3C2G, FGFR2, IRS2, PDE4B, GNG7
Growth hormone signaling	2.02	FLT3, CSHL1, PIK3C2G, FGFR2, CSH1/CSH2, IRS2, PRKCH
Gap junction signaling	1.95	GJA10, GRIA1, FLT3, GRIK3, GNAI1, PIK3C2G, FGFR2, IRS2, PRKCH, GRIK2, CTNNB1, GRIK1
Synaptic long term depression	1.94	GRM5, CACNA1I, CACNG6, GRIA1, GRID2, RYR3, GNAO1, CACNB4, GNAI1, PRKCH, CACNA1A
G-protein coupled receptor signaling	1.94	HTR5A, PDE10A, FLT3, PIK3C2G, GNAI1, FGFR2, DRD5, PDE4B, CREB5, CHRM3, GRM5, SHC1, GNAO1, IRS2, DUSP4
GABA receptor signaling	1.82	CACNA1I, CACNG6, KCNN3, GABRB3, CACNB4, SLC6A1, CACNA1A
Huntington’s disease signaling	1.68	GRM5, SHC1, HDAC4, IFT57, FLT3, GNG2, PIK3C2G, FGFR2, DNM3, IRS2, PRKCH, CREB5, GNG7
Neurotrophin/TRK signaling	1.55	SHC1, FLT3, PIK3C2G, FGFR2, IRS2, CREB5
α-adrenergic signaling	1.42	GNG2, GNAI1, PRKCH, PYGL, SLC8A1, GNG7
**Canonical pathways (validation)**	−Log (*p*-value)*	**Molecules**
CREB signaling in neurons	4.15	RAP2B, CACNG6, CACNA1H, PIK3R5, GNAI1, GNG7, GRM5, ADCY9, CACNA2D1, ADCY1, PRKAR1B, PIK3R6, ATF4, IRS2, CACNB2, ADCY8, GNAL, GRIA3
Endocannabinoid developing neuron pathway	4.02	RAP2B, PIK3R5, GNAI1, GNG7, ADCY9, ADCY1, PRKAR1B, PIK3R6, ATF4, IRS2, ADCY8, CTNNB1, GNAL
Calcium signaling	3.14	RAP2B, CACNG6, HDAC4, MYH9, MYH14, HDAC1, CACNA1H, TRPC7, CACNA2D1, MYH3, PRKAR1B, ATF4, CACNB2, CHRNA3, GRIA3
GABA receptor signaling	2.77	GABRG3, CACNG6, ADCY9, ADCY1, CACNA2D1, GABRA6, CACNA1H, CACNB2, ADCY8
G beta gamma signaling	2.6	RAP2B, CACNG6, ADCY1, CACNA2D1, GNAI1, PRKAR1B, CACNA1H, CACNB2, GNG7, GNAL
Gap junction signaling	2.56	RAP2B, GJA1, ACTB, PIK3R5, GNAI1, ADCY9, ADRB1, ADCY1, PRKAR1B, PIK3R6, IRS2, CTNNB1, ADCY8, GRIA3
Synaptic long term depression	2.18	RAP2B, GRM5, CACNG6, CACNA2D1, GNAI1, CACNA1H, CACNB2, PPP2R5C, PPP2R5E, NOS2, GNAL, GRIA3
Netrin signaling	1.94	CACNG6, CACNA2D1, PRKAR1B, CACNA1H, CACNB2, UNC5C
Notch signaling	1.66	MAML2, HES7, JAG1, PSEN1
Ephrin receptor signaling	1.48	ITGB1, RAP2B, EPHA6, SDCBP, SH2D3C, GNAI1, ATF4, EPHA3, GNG7, GNAL
Serotonin receptor signaling	1.48	ADCY9, SMOX, ADCY1, ADCY8
Axonal guidance signaling	1.37	RAP2B, ITGB1, BMP4, NRP2, ADAMTS20, PTCH1, PIK3R5, GNAI1, EPHA3, ROBO1, GNG7, EPHA6, SDCBP, WNT3A, PIK3R6, PRKAR1B, IRS2, BMP6, MMP17, GNAL, UNC5C

*Negative logarithm of the Fisher exact *p*-value indicating the probability that the described function is not enriched among the indicated genes based on the reference set of genes in the IPA knowledgebase.

**TABLE 3:** Nervous system functions enriched among DDE DMR-associated genes from discovery and validation data sets.

Nervous system development and functions (discovery)	*p*-value*	Molecules
Development of central nervous system	3.71E-06	ANKLE2, ASIC2, ATOH1, CNTN6, CNTNAP2, EML1, GRIK1, GSK3B, HGF, JARID2, MBP, MYO16, PAX6, PDGFC, PROX1, TRAPPC9
Synaptic transmission	1.22E-03	ASIC2, GRIA1, GRIK1, GRIK2, GRM5, MBP, NRG3, RIT2, SLC6A1, SYT1
Recognition of neurons	2.23E-03	NTM, OPCML
Outgrowth of neurites	3.11E-03	GFRA2, GSK3B, HGF, mir-124, SHC1, SLIT2, TGFA
Guidance of axons	3.88E-03	DOK5, GFRA2, IRS2, NRXN3, NTN4, SHC1, SLIT2, UNC5A
Generation of nervous tissue cell lines	7.17E-03	MYT1L, RMST
Outgrowth of axons	1.06E-02	HGF, SLIT2
Formation of brain	1.83E-02	CNTNAP2, EML1, GSK3B, HGF, MYO16, TRAPPC9
**Nervous system development and functions (validation)**	*p*-value*	**Molecules**
Recognition of neurons	2.50E-03	NTM, OPCML
Outgrowth of neurites	4.23E-03	BMP4, ITGA1, ITGB1, mir-10, TGFA, TIAM1, WNT3A
Proliferation of neuronal cells	5.08E-03	BMP4, ITGA1, ITGB1, JAG1, mir-10, TGFA, TIAM1, WNT3A
Neurotransmission	9.48E-03	CBLN1, DTNA, GPR176, GRM5, KCNQ1, MBP, MYH14, PSEN-1, SYT1
Quantity of neuroepithelial cells	1.18E-02	BMP4, BMP6

*Fisher exact *p*-value indicating the probability that the described function is not enriched among the indicated genes based on the reference set of genes in the IPA knowledgebase.

**FIGURE 8:**
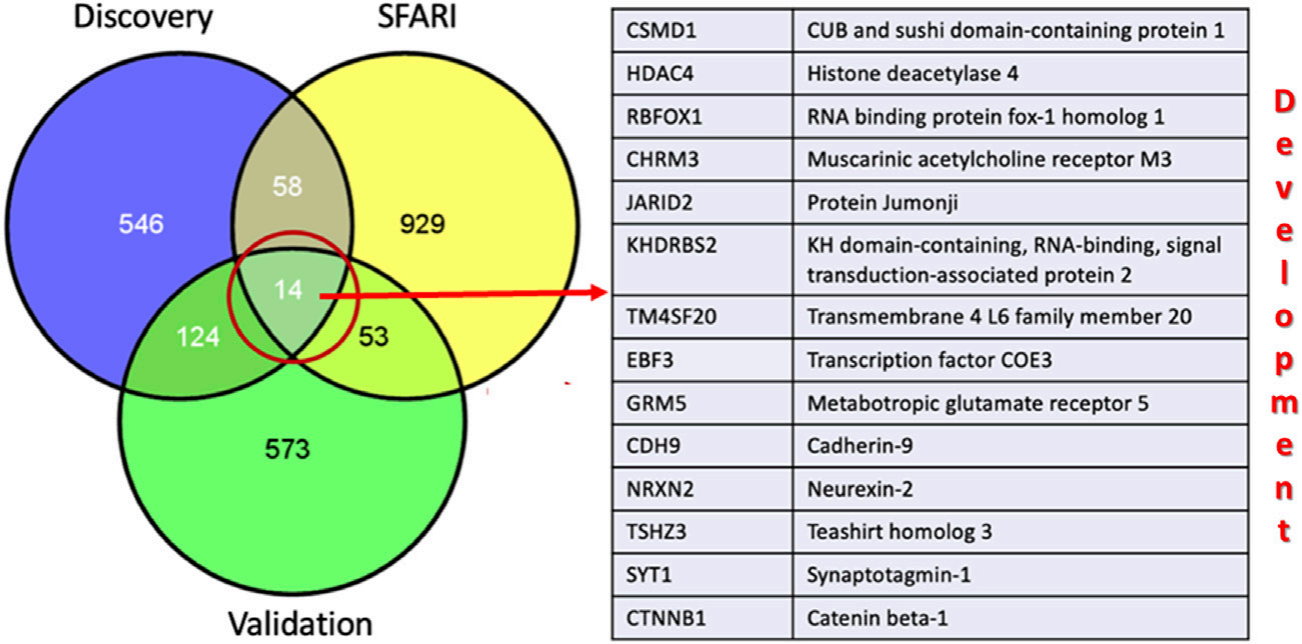
Overlap of DDE DMR-associated genes among the discovery and validation sets and those in the SFARI Gene database of ASD risk genes.

We employed hypergeometric analyses to investigate whether there was a significant overlap of DMR-associated genes between discovery and validation data sets. Moreover, because the gene ontology and pathway analyses implicated functions and pathways critical to neurodevelopment, we also determined the overlap of the DDE DMRs with genes in the SFARI Gene Database of over a thousand ASD risk genes identified primarily on the basis of genetic analyses. Figure 8 shows the overlap between DMR-associated genes from the discovery and validation analyses as well as the overlap between these DMR-associated genes and SFARI genes. Hypergeometric analyses showed that the 138 genes shared between the discovery and validation data sets represented significant overlap of DMRs between the two cohorts (upper cumulative Q-value = 4.08 × 10^−58^). Moreover, IPA analysis of the overlapping genes revealed significant over-representation of genes involved in nervous system development and function (Table 4). In addition, DMRs in both data sets were significantly enriched in SFARI genes with hypergeometric distribution Q-values for enrichment of 2.5 × 10^−7^ and 1.1 × 10^−5^ for the discovery and validation sets, respectively. Of the 138 overlapping genes between the discovery and validation cohorts, 14 genes that are also included in the SFARI Gene database are all involved in development.

**TABLE 4:** Nervous system functions enriched among the overlapping DDE DMR-associated genes between the discovery and validation data sets.

Nervous system development and functions (Overlap)	*p*-value*	Molecules
Development of central nervous system	5.19E-07	CTNNB1, EBF2, EBF3, FAT4, GRM5, IRS2, IRX6, JARID2, KHDRBS2, LHX2, MBP, NRXN2, PRDM16, R, ARB, TENM2, TFAP2C, ZBTB18
Sensory system development	1.35E-06	CTNNB1, EBF2, EBF3, FGF3, HS6ST1, IRS2, IRX6, LHX2, MAF, PRDM1, RARB, SLC4A3, TENM2, TGF, A
Formation of eye	1.14E-05	EBF2, EBF3, HS6ST1, IRS2, IRX6, LHX2, MAF, PRDM1, RARB, SLC4A3, TENM2, TGFA
Formation of brain	1.25E-05	CTNNB1, EBF2, EBF3, FAT4, GRM5, IRS2, IRX6, KHDRBS2, LHX2, PRDM16, RARB, TFAP2C, ZBTB18
Development of cerebral cortex	3.82E-05	CTNNB1, FAT4, GRM5, LHX2, PRDM16, TFAP2C, ZBTB18
Differentiation of type 2 OFF-cone bipolar cells	4.42E-05	EBF2, EBF3
Morphology of eye	6.82E-05	HS6ST1, IRS2, IRX6, LHX2, MAF, RARB, SLC4A3, TENM2, TGFA
Formation of olfactory receptor neurons	8.82E-05	EBF2, EBF3
Formation of olfactory receptor neurons	8.82E-05	EBF2, EBF3
Abnormal morphology of eye	1.17E-04	HS6ST1, IRS2, LHX2, MAF, RARB, SLC4A3, TENM2, TGFA
Differentiation of neurons	1.21E-04	CTNNB1, EBF2, EBF3, KLF6, LHX2, NKX2-5, SALL3, TGFA, ZBTB18, ZNF536
Recognition of neurons	1.47E-04	NTM, OPCML
Development of bipolar cells	2.19E-04	EBF2, PRDM1
Neurotransmission	2.35E-04	CHRM3, CTNNB1, GNAI1, GRM5, HDAC4, MBP, NRXN2, RARB, RASD2, SYT1
Development of sensory neurons	2.80E-04	CTNNB1, EBF2, EBF3
Formation of olfactory glomeruli	3.06E-04	EBF2, EBF3
Development of lens tissue	6.51E-04	MAF, TGFA
Morphology of central nervous system	7.71E-04	CTNNB1, EBF2, IRX6, KHDRBS2, LHX2, MBP, PRDM16, SLC4A3, TENM2, TGFA, ZBTB18
Binding of hippocampal neurons	7.94E-04	GRM5, INSR
Abnormal morphology of hypoglossal nerve	7.94E-04	RARB, SALL3
Sorting of axons	9.50E-04	CTNNB1, EBF2
Synaptic transmission	1.02E-03	CHRM3, CTNNB1, GNAI1, GRM5, MBP, NRXN2, RASD2, SYT1
Differentiation of amacrine cells	1.12E-03	EBF2, EBF3
Development of retinal pigment epithelium	1.30E-03	LHX2, RARB
Inhibitory postsynaptic current	1.34E-03	CHRM3, NRXN2, SYT1
Morphology of nervous system	1.51E-03	CTNNB1, EBF2, FGF3, GRM5, IRX6, KHDRBS2, LHX2, MBP, PRDM16, RARB, SALL3, SLC4A3, TENM2, TGFA, ZBTB18
Formation of forebrain	1.60E-03	CTNNB1, EBF2, EBF3, IRX6, LHX2, RARB
Accumulation of neuroglia	1.71E-03	CTNNB1, HS6ST1
Development of neurons	1.83E-03	CDH9, CTNNB1, EBF2, EBF3, GRM5, HS6ST1, IGSF21, IRX6, MBP, NRXN2, PRDM1, SDK2, ZBTB18
Formation of olfactory bulb	1.88E-03	EBF2, EBF3, LHX2
Proliferation of neural precursor cells	2.32E-03	CTNNB1, LHX2, RARB, ZBTB18
Size of brain	2.33E-03	CTNNB1, IRX6, KHDRBS2, PRDM16, TGFA
Abnormal morphology of iris	2.68E-03	RARB, TGFA
Abnormal morphology of cerebral neocortex	2.68E-03	LHX2, ZBTB18
Firing of brain cells	2.96E-03	GRM5, RBFOX1
Abnormal morphology of retina	3.41E-03	IRS2,LHX2, RARB, SLC4A3, TENM2
Abnormal morphology of vertebrae	3.55E-03	FAT4, FGF3, HDAC4, RARB
Exit from cell cycle progression of Schwann cells	3.86E-03	CTNNB1
Conduction of motor neurons	3.86E-03	EBF2
Development of tanycyte	3.86E-03	LHX2
Differentiation of inner hair cells	3.86E-03	CTNNB1
Differentiation of sensory progenitor cells	3.86E-03	CTNNB1
Differentiation of outer hair cells	3.86E-03	CTNNB1

*Fisher exact *p*-value indicating the probability that the described function is not enriched among the indicated genes based on the reference set of genes in the IPA knowledgebase.

### Pyrosequencing analyses of several differentially methylated region-associated autism spectrum disorder risk genes

**FIGURE 9:**
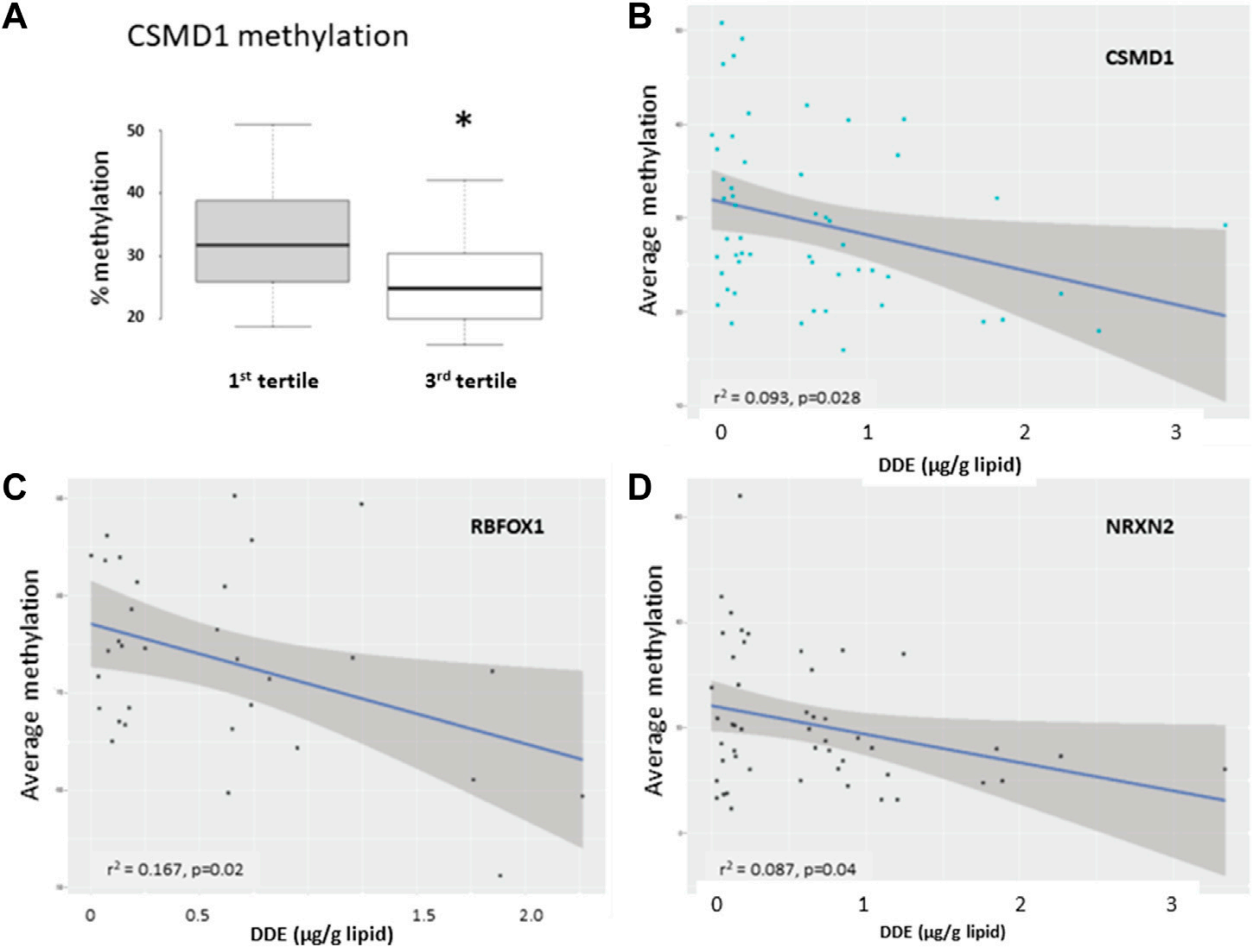
Results of pyrosequencing analyses of DMRs associated with *CSMD1* **(A,B)**, *RBFOX1* **(C)**, and *NRXN2* **(D)**. The box plot **(A)** shows differential methylation at a single CpG site in *CSMD1* while the graphs show the average methylation as a function of DDE serum concentration (μg/gm lipid) for *CSMD1*, *RBFOX1* (10 sites, discovery set only), and *NRXN2* (7 sites, all samples in both discovery and validation sets). R-squared (r^2^) and *p*-values for the correlation curves are shown.

Several DMRs were selected for pyrosequencing validation. These included the regions harboring known autism risk genes, *CSMD1*, *NRXN2*, and *RBFOX1*, all of which were found to be hypomethylated in both discovery and validation analyses. In addition, we included *PTPRN2* which, like *NRXN2*, was found to be hypomethylated in cord blood from the Faroese population as a function of level of DDE exposure (Leung et al., 2018). Also selected for pyrosequencing were two SNORDs (*SNORD115-30* and *SNORD115-37*) which are located in an imprinted region identified as significantly hypermethylated on a genome-wide level in this study as well as in a previous study on paternal sperm from men with an autistic child (Feinberg et al., 2015). Figure 9 shows the correlation curves for methyation level versus serum DDE exposure level for *CSMD1*, *NRXN2*, and *RBFOX1*. All of these genes showed a modest but statistically significant inverse correlation between methylation and DDE exposure levels. Consistent but not statistically significant increases in methylation at multiple CpG sites within *SNORD115-30* and *SNORD115-37* were observed in the samples with higher exposures (Table 5). *PTPRN2*, on the other hand, showed no correlation between DDE exposure and methylation levels as detected by pyrosequencing.

**TABLE 5:** Differential methylation of *SNORD115-30* and *SNORD115-37* by DDE tertile validated by pyrosequencing analyses.

SNORD115-30^a^								Average
Tertile	Pos. 1	Pos. 2	Pos. 3	Pos. 4	Pos. 5	Pos. 6	Pos. 7	All positions
First	28.98	30.76	34.31	33.49	37.26	44.31	30.56	34.24
Third		33.82	35.65	39.14	37.78	39.80	47.28	38.13
Difference (third-first)	4.84	4.89	4.82	4.30	2.54	2.97	2.89	3.89

SNORD115-37^b^					Average			
Tertile	Pos. 1	Pos. 2	Pos. 3	Pos. 4	All positions			
First	41.12	36.29	33.84	41.63	38.22			
Third	45.86	41.88	39.41	46.01	43.29		
Difference (third-first)	4.74	5.59	5.57	4.38	5.07		

a Methylation was quantified at 7 specific positions in the SNORD115-30 DMR.

b Methylation was quantified at 4 specific positions in the SNORD115-37 DMR.

### Comparison of differentially methylated region-associated genes from this study with those from autism spectrum disorder-related and unrelated methylation studies

The DDE DMR-associated genes in sperm were compared to differentially methylated genes in a variety of tissues from studies on ASD-associated methylation differences. These tissues included cord blood from newborns, a fraction of whom was later diagnosed with ASD (Mordaunt et al., 2020), sperm (Feinberg et al., 2015) and placenta (Zhu et al., 2019; Zhu et al., 2022) from parents of children with high risk for ASD, brain tissues from individuals with Dup15q syndrome that is often associated with ASD (Dunaway et al., 2016) as well as lymphoblastoid cell lines derived from individuals with a severe form of ASD (Hu et al., 2020).

Hypergeometric distribution analyses show that the DMR-associated genes from both the discovery and validation WGBS analyses overlapped significantly with those from ASD cord blood, paternal sperm, placenta, and Dup15q brain that also showed detectable PCB 95 exposures (Dunaway et al., 2016) (Table 6). DMR-associated genes from the discovery, but not validation, set also overlapped significantly with those from lymphoblastoid cell lines from ASD individuals in comparison to those of nonautistic siblings.

**TABLE 6:** Overlap among DDE DMR-associated genes and DMRs from different ASD studies and tissues.

Samples for comparison of DDE DMRs in sperm	Hypergeometric distribution Q-value	
(# DMR-associated genes)	Discovery (742)	Validation (763)
Cord blood from newborns later diagnosed with ASD^a^(2173)	2.96E-23	8.56E-10
Sperm (high ASD risk)^b^ (144)	1.22E-06	9.95E-05
Placenta (ASD outcome)^c^ (596)	1.56E-04	6.78E-05
Placenta (ASD outcome^d^ (134)	4.38E-05	5.65E-04
Brain (Dupl5q)^e^ (942)	1.90E-03	2.00E-03
Lymphoblastoid cell lines^f^ ^,^ ^g^ (827)	1.90E-02	8.00E-02
Pan-cancer^h^ ^,^ ^i^ (434)	7.34E-12	2.17E-09
Faroese cord blood (DDE associated)^j^ (1136)	0.608	0.55

a Mordaunt et al.(2020).

b Feinberg et al.(2015).

c Zhu et al.(2019).

d Zhu et al.(2022).

e Dunaway et al.(2016).

f Hu et al.(2020).

g Males only, ASD-severely language-impaired.

h Su et al.(2018).

i Hypermethylated canyon genes.

j Leung et al.(2018).

In addition, the DDE DMR-associated genes from both discovery and validation datasets significantly overlapped with those from pan-cancer studies (Su et al., 2018). Despite significant enrichment in DMRs from ASD cord blood, there was no significant overlap of DDE DMR-related genes with those in cord blood from the Faroese population that were also associated with DDE exposures (Leung et al., 2018). Interestingly, DMR-associated genes from both sperm (discovery set) and cord blood showed enrichment in multiple canonical pathways associated with neurological functions (Supplementary Table S4). Comparison of the DMR-associated genes from the ASD and Faroese cord blood studies showed no significant overlap (hypergeometric distribution Q = 0.84), but both sets of DMRs were highly enriched in genes on the X-chromosome. However, the X-linked genes from the ASD cord blood were predominantly found in females (Mordaunt et al., 2020), while those from the Faroese cord blood were exclusively male-specific (Leung et al., 2018). By comparison, there were relatively few X-linked DDE DMR-associated genes found in sperm from Faroese men.

## Discussion

### Methylation patterns of sperm DNA are associated with organochlorine 1,1-dichloro-2,2-bis(p-chlorophenyl)ethylene exposure levels

This study shows that the DNA methylation status in sperm may be influenced by lifelong exposure to environmentally derived persistent EDCs, such as DDE. The Faroese cohort used in this study is particularly exposed to higher than average levels of EDCs as a result of their natural diet which includes substantial amounts of pilot whale meat and blubber. Fatty tissues are reservoirs for lipophilic molecules, which include a wide variety of organochlorines, such as the long-lived DDE and PCBs. The high correlation between the levels of multiple organochlorines and DDE in serum (Figure 2) indicates that the DDE exposures employed in this study may be proxies for exposures to persistent organochlorines in general. Although some of these compounds, including the insecticide DDT (the parent compound of DDE) have now been banned for use, the long half-lives of such compounds and/or their breakdown products still pose risk of environmental exposures.

### Differentially methylated regions harbor genes enriched for nervous system development and function

Although there are hundreds of genes whose methylation status is associated with elevated exposure to DDE and other organochlorines, our functional and pathway analyses reveal that genes involved in nervous system development and function are the most significantly over-represented among DMRs from both the discovery and validation data sets as well as from the set of overlapping DMRs between data sets. Moreover, a significant number of these genes are also autism risk genes that are included in the SFARI Gene Database. Among the DMR-associated ASD risk genes validated by pyrosequencing are *CSMD1*, *NRXN2*, and *RBFOX1*. *CSMD1*, which encodes for CUB and Sushi multiple domains 1, is highly expressed in brain tissues where it has been associated with neuronal growth cone stabilization and neuritogenesis (Molenaar et al., 2012). Aside from being a risk gene for ASD (Hu et al., 2011; Cukier et al., 2014; Guo et al., 2017), it has also been implicated in schizophrenia, bipolar disorder, and post-traumatic stress disorder (Woo et al., 2017). *NRXN2* codes for neurexin 2, a brain-enriched cell adhesion molecule that has long been associated with ASD (Gauthier et al., 2011; Mohrmann et al., 2011; Dachtler et al., 2015). NRXN2 plays a role in early cortical synaptogenesis and axon guidance (Harkin et al., 2017). *RBFOX1* encodes for RNA binding fox-1 homolog 1, a neuron-specific splicing factor that has been implicated in many studies on ASD (Martin et al., 2007; Griswold et al., 2015; Bacchelli et al., 2020). Interestingly, *RBFOX1* was one of the top transcriptional targets of the orphan nuclear hormone receptor RORA which we found to be reduced in both lymphoblasts and brain tissues from individuals with ASD and regulated by sex hormones (Nguyen et al., 2010; Sarachana et al., 2011; Sarachana and Hu, 2013; Hu et al., 2015) as well as EDCs, including DDE and atrazine, an herbicide (Shu, Kocher, and Hu, unpublished data).

### Overlap of organochlorine 1,1-dichloro-2,2-bis(p-chlorophenyl)ethylene differentially methylated regions and those associated with autism spectrum disorder

A comparison of DDE DMRs from this study and those from studies of various tissues derived from individuals with ASD shows significant enrichment in DMRs associated with ASD (Table 6). Interestingly, the significance of the overlap between the DDE DMR-associated genes is highest in DMRs identified in cord blood from children diagnosed with ASD and lower in the more differentiated or immortalized tissues, i.e., brain and lymphoblasts, respectively. In the study on placental methylation, placentas of high-risk mothers with a child (or children) already diagnosed with ASD were obtained after the birth of a subsequent child for WGBS analysis (Schroeder et al., 2016). Subsequent analysis of DMRs from the placentas for ASD outcome revealed 596 nominally significant genes, of which two (*CYP2E1* and *IRS2*) reached genome-wide significance (Zhu et al., 2019). A more recent study on the placental methylome of a larger cohort of children who were diagnosed with ASD by 36 months (Zhu et al., 2022) further confirmed significant overlap with DDE DMR-associated genes in sperm. In the latter placental study, a novel brain regulatory region at 22q13.33 with bivalent chromatin was identified as being significantly hypomethylated. Interestingly, two genes in this region (*FAM19A5* and *BRD1*) overlap with the DDE DMR-associated genes from sperm. Furthermore, this region contains a copy number variant for ASD (Pinto et al., 2010) and a single nucleotide polymorphism that associates with severe language impairment in a GWAS analysis of subphenotypes of ASD (Hu et al., 2011).

With respect to sperm methylation in the context of ASD, semen samples from fathers of a child already diagnosed with ASD and those of neurotypical children were compared to identify DMRs (Feinberg et al., 2015). Noteworthy among the overlapping DMRs between this paternal sperm study and the DDE DMRs reported here is the extensive methylation differences in the noncoding *SNORD115* region on chromosome 15 that are further discussed below. It is also notable that DNA methylation differences between zona pellucida-bound sperm and manually selected sperm (typically used for intracytoplasmic sperm injection (ICSI) methods to treat infertility) were enriched for genes involved in ASD and neurogenesis, especially in regions with bivalent chromatin structure (Wang et al., 2021). These findings may explain the higher risk of ASD that is associated with ICSI (Kissin et al., 2015; Djuwantono et al., 2020), and further suggest a potential paternal contribution to ASD based on the sperm methylome. The present study describes a plausible environmental mechanism for introducing ASD-relevant methylation changes in sperm. Together, the placenta and sperm studies revealed DMR-associated genes related to ASD outcome or higher risk for ASD, respectively, in offspring of the high-risk parents in comparison to parents of neurotypical children, while the DMR-associated genes in ASD cord blood may be more directly related to ASD diagnosis in the individual.

### Endocrine disrupting compound-associated differential methylation also impacts non-coding regions of the genome

The altered methylation in a large block on chromosome 15 encompassing the non-coding *SNORD115* region is of particular interest inasmuch as this region was also found to be differentially methylated in the sperm of fathers with children exhibiting ASD in comparison to the sperm of fathers of unaffected children (Feinberg et al., 2015). However, the origins of such differences in sperm DNA methylation that are associated with ASD are unknown. The results from this study suggest that environmental exposures to certain EDCs may in part be responsible for alterations in the sperm methylome including the *SNORD115* region. This region is imprinted maternally and normally shows compact heterochromatin until its expression, which is exclusive to neurons. Intriguingly, the *SNORD115* locus is not expressed in any tissue besides the brain. It is notable that both the *SNORD115* and *SNORD116* noncoding loci were differentially methylated in the cross-cortical (prefrontal combined with temporal cortices) analyses of brain tissues from individuals with Dup15q syndrome but not from that of individuals with idiopathic ASD (Wong et al., 2019). In fact, there is a significant overlap of 38 DMRs (including *CSMD1* and 6 *SNORD115* variants) from this brain study (Wong et al., 2019) and the DDE DMRs identified in the discovery cohort from this study (hypergeometric distribution Q = 0.009). Although there were 32 shared DMRs with the validation data set, the overlap was not significant (Q = 0.138). These comparative analyses collectively suggest that DDE DMRs in sperm may be mirrored in various tissues (including brain) from individuals with ASD.

With respect to function, the *SNORD115* region on human chromosome 15q11-q13 contains a series of 48 highly conserved small nucleolar RNAs (snoRNAs) that participate in the modification of other non-coding RNAs and site-specific 2′-O-methylation of substrate RNAs (Galardi et al., 2002) as well as alternative splicing and RNA editing, especially of 5-HTR2C pre-mRNA (Cavaillé, 2017; Bratkovič et al., 2018; Raabe et al., 2019). In addition, a previous study also identified alterations at this locus in association with exposures to EDCs, albeit of a more transient (non-persistent) nature. Specifically, increased methylation was observed in the *SNORD115* locus in human fetal lung tissue of discontinued pregnancies of women exposed to BPA (Faulk et al., 2015). Although deletions in this genomic region are associated with Prader-Willi syndrome (PWS), loss of *SNORD115* alone is not sufficient to cause the disease (Bürger et al., 2002; Runte et al., 2005). Maternal duplications in the chr15q11-q13 region (also known as Dup15q syndrome) have also been associated with ASD as well as other developmental disorders, with some genes showing altered methylation status (Depienne et al., 2009; Scoles et al., 2011; Finucane et al., 2016). As mentioned earlier, Dup15q was shown to be a strong predictor of PCB 95 exposure (Mitchell et al., 2012). The Mitchell et al. study further showed that LINE-1 methylation was reduced in Dup15q and PWS samples but not idiopathic ASD, suggesting gene by environment interactions possibly mediated through epigenetic modifications in the genetically defined but not idiopathic forms of ASD. However, *SNORD115* genes were not specifically implicated in these Dup15q studies. The present study, coupled with that of Feinberg et al. (2015) on sperm from fathers of a child with ASD, reveals an additional epigenetic mechanism through which long-lived EDCs may mediate ASD-related changes in this region. Moreover, such changes in germline cells raise the possibility of transgenerational inheritance of phenotype as described in multiple animal studies.

### Potential significance of methylation changes in sperm cells

Previous studies on animal models have reported transgenerational effects of EDC exposures on disease and behavioral phenotypes, some of which were shown to be mediated by epigenetic changes in the germline (Anway and Skinner, 2008; Skinner et al., 2011; Manikkam et al., 2012; McBirney et al., 2017). Initial findings in rodents have shown that exposure to vinclozolin (an androgenic EDC) or methoxychlor (an estrogenic EDC) led to increased male infertility and related characteristics such as decreased sperm count in all subsequent generations, from F1 through F4 (Anway et al., 2005). In addition, when gestating F0 females were given various doses of a mix of EDCs during embryonic development, F1 and F3 generations exhibited increased total disease. Differentially methylated regions were found in sperm of the F3 generation that included promoters of genes associated with underlying diseases such as obesity, PCOS, and ovarian disease (Manikkam et al., 2013). These studies and others (McBirney et al., 2017) indicate that F0 exposure can lead to transgenerational effects on phenotypes that were associated with epigenetic changes in sperm that are specific to adult-onset disease.

The association between sperm methylation changes and levels of exposure to DDE presented in this study thus suggests a plausible mechanism for introducing epigenetic modifications in sperm DNA that are significantly over-represented in sperm from fathers of children with ASD (Table 6). Based on results from the animal studies mentioned above, these methylomic changes in human sperm, if transgenerational, may influence the phenotype of progeny with respect to a host of diseases, including ASD (Osumi and Tatehana, 2021). Moreover, as illustrated in Table 6, there is significant over-representation of the DDE DMR-associated genes in different tissues of individuals with ASD, implying that such methylomic patterns that derive from sperm may be mirrored in the brain and other tissues of individuals with ASD.

### Advantages and limitations of this study design and future directions

A primary advantage of this study on sperm from men with varying levels of exposure to DDE is that the Faroese population is considered a “genetic isolate,” thus reducing genetic heterogeneity that is often a major challenge in epigenetic studies. In addition, all the semen donors were on average 25.44 (±0.41) years old, thus eliminating confounding results based on age which has been shown previously to influence methylation patterns (38,39). Another advantage of this cohort is the natural Faroese diet, rich in pilot whale meat and blubber, that exposes a fraction of the population to higher than average levels of fat-soluble long-lived organochlorines and other POPs. While we show differences in methylation patterns in sperm from men with high versus low exposures to DDE that were highly correlated with the sum of the four most prevalent PCB congeners (Figure 2), an obvious limitation of this study is the total number of samples included. This limitation was the result of the limited availability and volume of semen samples with a sperm count of 20 × 10^6^/ml (≤100 μl was provided for this study) to allow for isolation of sufficient amounts of DNA for the bisulfite sequencing analyses followed by pyrosequencing validation of selected gene regions. Despite the limitation in sample size, by dividing the samples into discovery and validation sets for the WGBS analyses, we were able to show a significant overlap of 138 DMRs between the two experimental cohorts. In addition, there were no completely unexposed samples for comparisons. Because EDCs are known to exhibit non-monotonic dose-response curves (Vandenberg et al., 2012), we cannot rule out the possibility that the lowest exposures examined here may show even greater methylation differences relative to unexposed controls than the differences between the first and third exposure tertiles included in this study. Moreover, because spermatocytes are transcriptionally inactive, we were not able to correlate methylation changes with changes in gene expression in the same tissues. Additionally, we could not correlate EDC exposure with ASD risk in offspring, as there was no information on children (if any) of the young men in this Faroese cohort. While the prevalence of ASD in the Faroe Islands appears to be comparable to that of other western countries at 0.56% in 2002 (Ellefsen et al., 2007), it has been reported that neurobehavioral deficits in a Faroese birth cohort of 7-year-old children are associated with prenatal exposures to organochlorine neurotoxicants in seafood as measured in umbilical cord tissue (Grandjean et al., 2001). Thus, it is possible that a study of children of fathers stratified according to levels of EDC exposures might reveal an association between high EDC exposures and ASD in their offspring.

Aside from investigating the possible relationships between organochlorine exposures, sperm DNA methylation profiles, and neurodevelopmental disorders, the availability of this publicly accessible methylation data on DDE/organochlorine-associated changes in the sperm methylome of exposed individuals will provide a valuable resource for studies on other diseases and conditions, such as cancer, obesity, diabetes, and infertility, which have also been linked to environmental exposures. Indeed, the data in Table 6 show highly significant over-representation of pan-cancer genes among those associated with DDE DMRs. The overlap with cancer genes is not surprising since organochlorine exposures are well-known risk factors for cancer; thus, the current study reveals exposure-associated epigenetic changes in DMR-associated genes that may also increase cancer risk (Dorgan et al., 1999; Jaga and Dharmani, 2005; Purdue et al., 2007). Interestingly, cancer and ASD share many risk genes and pathways (Crawley et al., 2016), some of which may be influenced by environmental factors.

Future studies should include additional cohorts sampled longitudinally to monitor temporal changes in individual sperm methylation levels as a function of cumulative EDC exposures as well as expanded concentration levels of DDE and other POPs. Moreover, it is unknown whether the DNA methylation differences noted in this study arose from direct exposure of the individual sperm donors to EDCs or from ancestral exposures that led to the inheritance of specific DNA methylation patterns in the donors’ sperm. Thus, future studies would also benefit from the analyses of sperm DNA samples coupled with measured EDC exposure levels within families (e.g., father, son, grandson) to determine potential heritability of the sperm methylome in the context of environmental exposures. Finally, given the association between exposure levels of DDE and altered methylation of many neurodevelopmental genes, it will also be of interest to investigate the relationships between DDE exposures of the semen donors, epigenetic changes in sperm, and the health outcomes of their children.

## Conclusion

This study shows that elevated exposure to DDE, one of a class of persistent organochlorines, is associated with genome-wide differential DNA methylation patterns in sperm when compared against the lowest exposure levels. The DMRs are enriched for genes involved in many biological processes, including neurological functions and pathways impacted by neurodevelopmental disorders. While not establishing a causal relationship between EDC exposures and the disorders *per se*, this study supports a link between environmental EDC exposures and epigenetic changes in germ cells that may impact the regulation of many genes associated with disease phenotypes, including ASD. Studies involving animal models have shown that DNA methylation patterns as well as associated phenotypes or diseases can be stably and heritably transmitted through the germline in several generations of offspring, specifically in relation to EDC exposure. If transgenerational epigenetic transmission can be demonstrated in humans, our results would suggest that heritable methylomic patterns in sperm may contribute to disease phenotypes, including ASD, in human progeny.

## Data Availability

The datasets presented in this study can be found in online repositories. The names of the repository/repositories and accession number(s) can be found below: https://www.ncbi.nlm.nih.gov/geo/, GSE165915.

## Supplementary material

SUPPLEMENTARY TABLE S1 DMRs identified in the discovery set.

SUPPLEMENTARY TABLE S2 DMRs identified in the validation set.

SUPPLEMENTARY TABLE S3 Summary of demographic and EDC data for sperm donors in each exposure tertile.

SUPPLEMENTARY TABLE S4 Canonical pathways enriched among overlapping DDE DMR-associated genes be-tween Faroese sperm and cord blood.

SUPPLEMENTARY FIGURE S1 Annotated genomic sites/regions among DMRs in discovery and validation sets.

SUPPLEMENTARY FIGURE S1 Primer sequences and PCR conditions for pyrosequencing analyses.
