# Supplementary material for: Elevated exposures to persistent endocrine disrupting compounds impact the sperm methylome in regions associated with autism spectrum disorder
Source: Front Genet. 2022 Aug 11;13:929471. doi: 10.3389/fgene.2022.929471 (PMC9403863; doi:10.3389/fgene.2022.929471)
Supplement: Supplementary file 2 [file Table4.pdf]

Table S4. Canonical pathways enriched among overlapping DDE DMR-associated genes between Faroese sperm and cord blood.

Canonical Pathways	-log(p-value)*	Molecules
Netrin Signaling	3.53	CACNA1I,NCK2,UNC5A
GABA Receptor Signaling	3.06	CACNA1I,GABRB3,KCNN3
Circadian Rhythm Signaling	2.71	CREB5,VIP
CREB Signaling in Neurons	2.02	CACNA1I,CREB5,GRIK2
Axonal Guidance Signaling	1.82	NCK2,NTN4,NTNG2,UNC5A
Cellular Effects of Sildenafil (Viagra)	1.58	KCNN3,PDE4B
GP6 Signaling Pathway	1.55	COL25A1,FGA
Corticotropin Releasing Hormone Signaling	1.51	CACNA1I,CREB5
Extrinsic Prothrombin Activation Pathway	1.5	FGA
Gustation Pathway	1.44	CACNA1I,PDE4B
Methionine Degradation I (to Homocysteine)	1.41	MGMT
Protein Kinase A Signaling	1.39	CREB5,PDE4B,PTPRS
Cysteine Biosynthesis III (mammalia)	1.37	MGMT
GNRH Signaling	1.35	CACNA1I,CREB5
Ephrin Receptor Signaling	1.31	CREB5,NCK2

*Negative logarithm of the Fisher exact p-value indicating the probability that the described pathway is not enriched among the indicated genes based on the reference set of genes in the IPA Knowledgebase
