# Supplementary figures and images for: Elevated exposures to persistent endocrine disrupting compounds impact the sperm methylome in regions associated with autism spectrum disorder
Source: Front Genet. 2022 Aug 11;13:929471. doi: 10.3389/fgene.2022.929471 (PMC9403863; doi:10.3389/fgene.2022.929471)

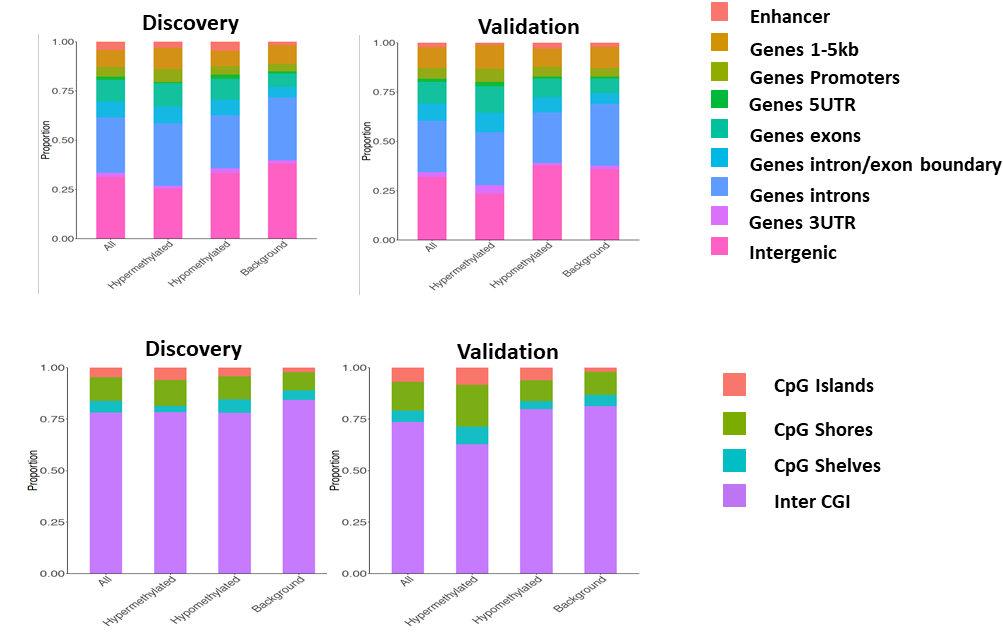

Supplement: Supplementary file 4 [file Image1.TIF]
