# Supplementary material for: Elevated exposures to persistent endocrine disrupting compounds impact the sperm methylome in regions associated with autism spectrum disorder
Source: Front Genet. 2022 Aug 11;13:929471. doi: 10.3389/fgene.2022.929471 (PMC9403863; doi:10.3389/fgene.2022.929471)
Supplement: Supplementary file 5 [file DataSheet1.pdf]

Primer sequences and PCR assay conditions for pyrosequencing analyses

CSMD1

CSMD1-assay1-R1	CGCCAGGGTTTTCCCAGTCACGACACCATAACAAAACCCCCTCTCTAC
CSMD1-assay1-S1	GTTGGGGTTATAGGTATATATTA

Use S1 and R1 to amplify a 77bp amplicon. Use S1 as a sequencing primer to analyze two CpG sites.

CSMD1-assay1(S1-R1) PCR assay

Total Volume (ul)	25
dH_2O	19.25
10*BF (HOT STAR)	2.5
10mM dNTP	0.5
10uM M13-Biotin Universal Primer	0.5
10uM Primer-S1	0.5
10uM Primer-R1	0.5
HotStar (Qiagen)	0.25
Bis-DNA	1
	25

PCR2:"60 to 55" on Bio Rad (RLT2632)

Cycle 1: (1X)	Step 1	:95C for 15:00
Cycle 2: (10X)	Step 1	:94C for 00:20
	Step 2	:60C for 00:20
Decrease Temp after cycle 1 by 0.5C every 1 cycle		
	Step 3	:72C for 00:20
Cycle 3: (40X)	Step 1	:94C for 00:20
	Step 2	:55C for 00:20
	Step 3	:72C for 00:20
Cycle 4: (1X)	Step 1	:72C for 7:00
Cycle 5: (1X)	Step 1	:12C for ~

CSMD1-assay1(S1-R1): 77bp(without M13)

NRXN1

NRXN2-F1	GGGAGGTTGGAGTTAAAGATTT
NRXN2-R1	CGCCAGGGTTTTCCCAGTCACGACCTACCTTCTACCCCTTCCTCA
NRXN2-S1	GGTTGGAGTTAAAGATTTT

Use F1 and R1 to amplify a 143bp amplicon. Use S1 as a sequencing primer to analyze nine CpG sites.

NRXN2 PCR assay

Total Volume (ul)	25
dH_2O	18.65
10*BF (HOT STAR)	2.5
25mM $MgCl_2$	1

10mM dNTP	0.5
10uM M13-Biotin Universal Primer	0.5
10uM Primer-F	0.5
10uM Primer-R	0.1
HotStar (Qiagen)	0.25
Bis-DNA	1
	25

PCR2:"60 to 55" on Bio Rad (RLT2631, 2632)

Cycle 1: (1X)	Step 1	:95C for 15:00
Cycle 2: (10X)	Step 1	:94C for 00:20
	Step 2	:60C for 00:20
Decrease Temp after cycle 1 by 0.5C every 1 cycle		
	Step 3	:72C for 00:20
Cycle 3: (40X)	Step 1	:94C for 00:20
	Step 2	:55C for 00:20
	Step 3	:72C for 00:20
Cycle 4: (1X)	Step 1	:72C for 7:00
Cycle 5: (1X)	Step 1	:12C for ~

NRXN2: 178bp(without M13)

PTPRN2

PTPRN2-F1	AGTGGGTTTTTTGTGGAAGTAAT
PTPRN2-R1	CGCCAGGGTTTTCCCAGTCACGACAAACCTAAATATACATAACTAAAACTACCT
PTPRN2-S1	AATATTTTTGTGAAAGGAG

Use F1 and R1 to amplify a 280bp amplicon. Use S1 as a sequencing primer to analyze 10 CpG sites.

PTPRN2 PCR assay

Total Volume (ul)	25
dH_2O	18.65
10*BF (HOT STAR)	2.5
25mM $MgCl_2$	1
10mM dNTP	0.5
10uM M13-Biotin Universal Primer	0.5
10uM Primer-F	0.5
10uM Primer-R	0.1
HotStar	0.25
Bis-DNA	1
	25

PCR2:"60 to 55" on Bio Rad (RLT2631)

Cycle 1: (1X)	Step 1	:95C for 15:00
Cycle 2: (10X)	Step 1	:94C for 00:20
	Step 2	:60C for 00:20
Decrease Temp after cycle 1 by 0.5C every 1 cycle		
	Step 3	:72C for 00:20

Cycle 3: (40X)	Step 1	:94C for 00:20
	Step 2	:55C for 00:20
	Step 3	:72C for 00:20
Cycle 4: (1X)	Step 1	:72C for 7:00
Cycle 5: (1X)	Step 1	:12C for ~

PTPRN2: 280bp(without M13)

RBFOX1 (32 Discovery samples)

RBFOX1-4A1-F7	AGAGAATTTTTAATGAGGGTAGGT
RBFOX1-4A1-R5	CGCCAGGGTTTTCCCAGTCACGACAAATTTTCTAAATTCAAAACCTCCTACAA
RBFOX1-4A1-S9	GGATGTTTTTGTTTTTTTGTTTAA
RBFOX1-4A2-S1	GTATAGAGGAGTATGGTT

Use F7 and R5 to amplify a 238bp region. Use F7 as a sequencing primer to analyze five CpG sites. Use S1 to analyze two CpG sites. Use S9 to analyze four CpG sites.

RBFOX1-4A1(F7-R5) PCR assay

Total Volume (ul)	25
dH_2O	18.25
10*BF (HOT STAR)	2.5
25mM $MgCl_2$	1
10mM dNTP	0.5
10uM M13-Biotin Universal Primer	0.5
10uM Primer-F7	0.5
10uM Primer-R5	0.5
HotStar (Qiagen)	0.25
Bis-DNA	1
	25

PCR2:"60 to 55" on Bio Rad (RLT2632)

Cycle 1: (1X)	Step 1	:95C for 15:00
Cycle 2: (10X)	Step 1	:94C for 00:20
	Step 2	:60C for 00:20
Decrease Temp after cycle 1 by 0.5C every 1 cycle		
	Step 3	:72C for 00:20
Cycle 3: (40X)	Step 1	:94C for 00:20
	Step 2	:55C for 00:20
	Step 3	:72C for 00:20
Cycle 4: (1X)	Step 1	:72C for 7:00
Cycle 5: (1X)	Step 1	:12C for ~

RBFOX1-4A1(F7-R5): 238bp(without M13)

SNORD115-30

SNORD115-30-A1-F1	ATTGATGGGTATAGGTGAGTGTA
SNORD115-30-A1-R1	CGCCAGGGTTTTCCCAGTCACGACAAACTTCAAAAAAAATATCCCCCTTA

SNORD115-30-A1-S1	GGGTTATGAGTTAGGT
SNORD115-30-A1-S2	AGGTATAGGTTTTTTAGGAG

Use F1 and R1 to amplify a 199bp amplicon. Use S1 as a sequencing primer to analyze the first two CpG sites. Use S2 to analyze the next two CpG sites.

SNORD115-30-A1 PCR assay

Total Volume (ul)	25
dH_2O	18.65
10*BF (HOT STAR)	2.5
25mM $MgCl_2$	1
10mM dNTP	0.5
10uM M13-Biotin Universal Primer	0.5
10uM Primer-F	0.5
10uM Primer-R	0.1
HotStar (Qiagen)	0.25
Bis-DNA	1
	25

PCR2:"60 to 55" on Bio Rad (RLT2631, 2632)

Cycle 1: (1X)	Step 1	:95C for 15:00
Cycle 2: (10X)	Step 1	:94C for 00:20
	Step 2	:60C for 00:20
Decrease Temp after cycle 1 by 0.5C every 1 cycle		
	Step 3	:72C for 00:20
Cycle 3: (40X)	Step 1	:94C for 00:20
	Step 2	:55C for 00:20
	Step 3	:72C for 00:20
Cycle 4: (1X)	Step 1	:72C for 7:00
Cycle 5: (1X)	Step 1	:12C for ~

SNORD115-30-A1: 199bp(without M13)

SNORD115-30-A2 PCR assay

Total Volume (ul)	25
dH_2O	18.25
10*BF (HOT STAR)	2.5
25mM $MgCl_2$	1
10mM dNTP	0.5
10uM M13-Biotin Universal Primer	0.5
10uM Primer-F	0.5
10uM Primer-R	0.5
HotStar	0.25
Bis-DNA	1
	25

PCR3: "55 to 50" on Bio Rad (RLT2632)

Cycle 1: (1X)	Step 1	:95C for 15:00
Cycle 2: (10X)	Step 1	:94C for 00:20
	Step 2	:55C for 00:20

Decrease Temp after cycle 1 by 0.5C every 1 cycle

	Step 3	:72C for 00:20
Cycle 3: (40X)	Step 1	:94C for 00:20
	Step 2	:50C for 00:20
	Step 3	:72C for 00:20
Cycle 4: (1X)	Step 1	:72C for 7:00
Cycle 5: (1X)	Step 1	:12C for ~

SNORD115-30-A2: 80bp(without M13)

SNORD115-37

SNORD115-37-F1	TTTTGGTTTTGAGAGGTATGGGTTAGAG
SNORD115-37-R3	CGCCAGGGTTTTCCCAGTCACGACACCATCCCTCCTAATAAATAACCT
SNORD115-37-S2	GGGTTATGAGTGAGGT

Use F1 and R3 to amplify a 143bp amplicon. Use S2 as a sequencing primer to analyze four CpG sites.

SNORD115-37 PCR assay

Total Volume (ul)	25
dH_2O	18.25
10*BF (HOT STAR)	2.5
25mM $MgCl_2$	1
10mM dNTP	0.5
10uM M13-Biotin Universal Primer	0.5
10uM Primer-F	0.5
10uM Primer-R	0.5
HotStar	0.25
Bis-DNA	1
	25

PCR1:"65 to60" on Bio Rad (RLT2631)

Cycle 1: (1X)	Step 1	:95C for 15:00
Cycle 2: (10X)	Step 1	:94C for 00:20
	Step 2	:65C for 00:20

Decrease Temp after cycle 1 by 0.5C every 1 cycle

	Step 3	:72C for 00:20
Cycle 3: (40X)	Step 1	:94C for 00:20
	Step 2	:60C for 00:20
	Step 3	:72C for 00:20
Cycle 4: (1X)	Step 1	:72C for 7:00
Cycle 5: (1X)	Step 1	:12C for ~

SNORD115-37: 143bp(without M13)
